# The effect of Orem-based self-care education on improving self-care ability of patients undergoing chemotherapy: a randomized clinical trial

**DOI:** 10.1186/s12885-022-09881-x

**Published:** 2022-07-15

**Authors:** Tayebeh Rakhshani, Siamak Najafi, Fakhry Javady, Alireza Taghian dasht bozorg, Fatemeh Mohammadkhah, Ali Khani Jeihooni

**Affiliations:** 1 Nutrition Research Center, Department of Public Health, School of Health, Shiraz University of Medical Sciences, Shiraz, Iran; 2 Department of Internal Medicine, School of Medicine, Fasa University of Medical Sciences, Fasa, Iran; 3 Department of Nursing, School of Nursing, Ahvaz Branch Islamic Azad University, Ahvaz, Iran; 4 Department of Community health, child nursing and aging, Ramsar School of Nursing, Babol University of Medical Sciences, Babol, Iran

**Keywords:** Cancer, Education, Orem’s self care, Chemotherapy

## Abstract

**Background:**

Cancer is a frightening disease. Therefore, the care of cancer patients is very complex. This study aimed to investigate the effect of the education based on Orem’s self-care model on self-care abilities of the patients undergoing chemotherapy in Shafa Hospital in Ahvaz city, Iran.

**Methods:**

This randomized clinical trial was carried out in 2018 on 100 cancer patients undergoing chemotherapy, who referred to Shafa Hospital in Ahvaz city, Iran. The sampling method was simple and the subjects were randomly divided into two experimental and control groups (50 in the experimental and 50 in the control group). The study outcome was the self-care ability of the patients measured before and 2 months after the intervention by the control and experimental groups. The educational intervention consisted of five 60-minute sessions (one session per week) held as educational and counseling ones through group, face-to-face and individual training based on the identified needs of the patients in the experimental group at Shafa Hospital Chemotherapy Department. To collect data, two questionnaires [the assess and identify the conceptual pattern of Orem questionnaire and the Self-care capacity assessment (ESCI)]were used. Statistical data were entered into SPSS software version 20 and analyzed by chi-square, independent t-test and paired t-test.

**Results:**

The mean and standard deviation of age was 35.06 ± 14.51 in the control group and 31.72 ± 15.01 in the experimental group. The results of the independent t-test showed that before the educational intervention, there was no significant difference between the two groups in terms of the mean self-care (*P* = 0.38). But after the intervention, a significant difference was found between the mean self-care scores of the experimental and control groups (*P* = 0.001).

**Conclusion:**

Application of Orem’s self-care model led to increased self-care ability of the cancer patients undergoing chemotherapy. Therefore, it is recommended that this model be included in the routine programs of chemotherapy departments.

**Trial registration:**

IRCT registration number: IRCT20160418027449N6.

Registration date: 01/05/2019.

## Background

Among the primary causes of death in both developed and developing countries, cancer is particularly prevalent in Iran [1], A total of 19.3 million new cancer cases (excluding nonmelanoma skin cancer) and more than 10.0 million new cancer deaths (9.9 million excluding nonmelanoma skin cancer) are expected to occur worldwide in 2020 (excluding nonmelanoma skin cancer) [2]. Cancer is the second leading cause of death in the globe, accounting for an estimated 9.6 million fatalities, or one in every six deaths, in 2018, according to estimates. Every year, more than 3.7 million new instances of cancer and 1.9 million deaths occur in Europe, making it the second biggest cause of death and sickness on the continent. Cancer was responsible for 8.2 million deaths globally in 2012, accounting for around 13% of all fatalities [3]. Iran’s population is expected to reach 85 million people by 2020, according to current estimates. According to the worldwide cancer observatory, the incidence of all cancer types in Iran was 70,704 in men and 60,484 in women, for a total of 131,191 malignancies in both sexes, according to the Global Cancer Observatory [1]. Also in the same year, 79.139 people lost their lives to cancer, with 46,436 men and 32,700 women succumbing to their disease. There were 319, 740 cases reported for both sexes over a 5-year period, including 161, 810 males and 157, 790 females. All cancer categories, with the exception of non-melanoma cancer, had an age-standardized incidence rate (ASR) of 165. 0 per 100,000 for males and 139. 0 per 100,000 for females in 2010 [4].. Apart from mortality, cancer causes incapacity and a myriad of bodily, emotional, and psychological concerns for those who are afflicted by it as well as for their loved ones [5]. Cancer diagnosis and treatment is a highly stressful event that frequently results in severe psychological suffering for both the patient and those around him. Cancer diagnosis and treatment is a highly stressful event that frequently results in severe psychological suffering for the patient and those around him. Psychiatric disorders frequently have a detrimental impact on treatment, adherence to treatment, quality of life, pain, and even the survival of the patient [6]. Cancer treatment methods, including chemotherapy, are associated with short- or long-term complications that endanger the patients’ quality of life. Patients receiving chemotherapy need active measures to control the adverse effects of treatment, and depending on the severity of the side effects, they may sometimes require to be admitted and receive the required care by the care-providing teams [7]. On the other hand, as cancer is a chronic disease, it is necessary for the patients to cooperated with the care teams in all stages of disease control and treatment and be able to perform self-care activities [8]. Currently, the best management approach to this chronic condition is self-care, which refers to patients’ involvement in self-observing, recognizing, and labeling symptoms and judging their severity, assessing, and adopting treatment choices, and evaluating the efficiency of self-care [9]. Self-care behavior is influenced by the set of skills and knowledge that a person uses to take action. Self-care measures include prevention, alleviation of pain, definitive treatment or control of diseases and condition of life-threatening, health and well-being. Self-care activities include seeking and participating in medical care, nursing, and other forms of health care. In case of illness or change in health status, self-care requires seeking medical services, referring to a physician and performing prescribed services or reviewing periods of health status [10] Therefore, nurses must teach the patients and clients how to resolve their problems and make decisions. The education needs to be proportionate to their problems and evolutionary level as well [11]. Self-care needs are related to the stage of human development. This means that these needs are commensurate with the stages of the life cycle in which the person is living. Life cycle stages are: intrauterine life and the birth process, Newborn, infancy, childhood and adolescence, adulthood, old age and pregnancy. A person’s self-care capacity in his development cycle from childhood to old age is located in different parts of a spectrum and its amount varies depending on the health status, factors affecting the educability and life experiences of the person because these are the factors that enable the person. To learn, to be exposed to cultural influences and to use the resources needed in his daily life [12]. One of the main pillars of self-care improvement is patient education. On the other hand, doing self-care requires self-healing abilities (ability of drug treatment in cancer patients [13].

In order to provide appropriate patient education, their educational needs should be taken into consideration and appropriate nursing care models have to be used. Orem’s self-care model is one of the most comprehensive self-care theories that provides an appropriate clinical guideline for planning and implementing self-care principles [14]. The self-care concept was introduced as an element of nursing theory by Orem [9]. According to Orem’s model, the goal of self-care is to achieve the following goals: supporting the life process and promoting normal functioning; maintaining normal growth, development and maturity; preventing, controlling and treating disease processes and injuries; preventing or compensating for disabilities; and promoting good feelings [15]. In fact, Orem’s model aims to encourage clients to take care of themselves, and the role of nurses is to assess the need for self-care and to determine the self-care power as well as the presence or absence of self-care deficit in chronic patients [16, 17]. Studies on the quality of life of cancer patients undergoing chemotherapy showed that the mean dimensions of quality of life after chemotherapy declined significantly compared to pre-treatment and could put the patients’ quality of life at risk in short- or long-term [18]. In their study, Gokal et al. examined the role of self-care education in the health status of the breast cancer patients undergoing chemotherapy, and reported that self-care enhanced mental health in those patients [19]. In another study, Zhang et al. emphasized the importance of self-care in cancer patients undergoing chemotherapy [20]. Empowering patients in the field of self-care helps to promote health and patients’ better understanding of the disease, better management of treatment side effects and control of symptoms, and improve quality of life [21]. Research on self-care in cancer patients has focused on the quantity and quality of their self-care behaviors and less on the self-care ability of these patients. Given the need to identify and take measures to control the debilitating problems of chemotherapy and improve their self-care capacity by the treatment team is strongly felt. It is important to motivate these patients to self-care and to plan based on this potential. Increasing the self-care ability of chemotherapy patients can lead to self-fulfillment. In view of the above, there is a need to review and research the design and selection of the newest and at the same time the most effective self-care program based on the needs of these patients to control their problems and increase their self-care capacity. On the other hand, there are some of studies on Orem’s self-care model for cancer patients, focusing only on the quantity and quality of their self-care behaviors [21–23], and less attention has been paid to improving the patients’ self-care abilities. Also, given that the ultimate goal of chemotherapy is to enhance the patients’ quality of life and health status by empowering them to perform self-care behaviors, implementation of educational programs for these patients can serve as a guide for macro-level planning and help healthcare providers to do more extended interventions. Hence, this study aimed to investigate the effect of the education based on Orem’s self-care model on self-care abilities of the patients undergoing chemotherapy in Shafa Hospital in Ahvaz city, Iran.

## Materials and methods

### Study design and participants

This randomized clinical trial was carried out in Ahvaz city, Iran in 2018. The population consisted of the cancer patients referred to the chemotherapy center in Shafa Hospital in Ahvaz city, Iran. The inclusion criteria were as follows: definitive cancer diagnosis by an oncologist, lack of pain and fatigue and being ready to participate in the study, lack of severe vision and hearing impairment, being in the age range determined (13–59 years old), not having medical education / not being in the medical or paramedical groups, being able to read and write, and not having received formal education in self-care against the side effects of chemotherapy. The exclusion criteria were unwillingness to participate in the research project, failure to follow self-care activities, and having pain and fatigue during the education period. In order to collect the study data, an observation and a questionnaire were used. The participants answered the questions with a self- report (based on the presence or absence of pain, the presence or absence of fatigue, etc.) and the questions answered have been reviewed and analyzed by Researchers.

### Sample size and sampling method

Considering the following formula at 95% confidence level and 90% test power,

sample size was estimated based on a previous study by karbaschi et al. [24] in which the mean ± SD of qulaity of life in ntervention group before and after intervention equale 68.81 ± 16.26 and 81.62 ± 11.1 respectively, the sample size stimated 26 person in each group. However 50 subjects were recruited in each group to compensate the possible attrition.

$${n}_1={n}_2=\frac{\left({S}_1^2+{S}_2^2\right)+{\left({Z}_{1-\frac{a}{2}}+{Z}_{1-\beta}\right)}^2}{{\left({\overline{X}}_1-{\overline{X}}_2\right)}^2}$$

The sampling method was purpose-based in that after referring to the cancer center in Shafa Hospital in Ahvaz city, Iran., patients who were eligible to enter the study were selected (100 people). The method of allocating the samples to the experimental and control groups was simple random method, so that the first person was randomly assigned to the experimental group by lot and the next person was placed in the control group and The routine continued by the end of the sample.

### Intervention

#### Experimental group

To design the educational intervention, Orem’s conceptual pattern recognition questionnaire was first completed by the participants. Then, the educational intervention was done on the participants’ weaknesses (chemotherapy complications, fatigue caused by chemotherapy, low self-confidence, high depression). After identifying the weaknesses, the researcher determined the limitations, shortcomings, disabilities and abilities, available resources, and study objectives based on the type of patient needs. In the intervention phase and with regard to the goals of education-based self-care programs, the experimental group received education on pain control during chemotherapy, mouth ulcers, weight loss, nausea and vomiting, constipation, anorexia, flatulence, food intolerance, hair loss, insomnia, and psychiatric disorders such as depression and lack of self-confidence.

The educational intervention consisted of five 60-minute sessions (one session per week) held as educational and counseling ones through group, face-to-face and individual training based on the identified needs of the patients in the experimental group at Shafa Hospital Chemotherapy Department (Table 1 and Table 2).

**Table 1:** Frequency distribution of Orem training needs in the intervention group

Patient educational needs	Title	Percentage
Reduce the number of blood cells in the body	nose bleeding	3
Changes in the digestive system	Weight Loss	25
	Mouth ulcers	31
	nausea and vomiting	31
	Blowing	20
	Anorexia	63
	Constipation	36
	Diarrhea	11
	Tooth Decay	9
Skin and nail changes	hair loss	36
	Darkening of the skin	26
Nervous system changes	Weakness	9
	Vertigo	10
	Depression	15
	Low self-confidence	24
Sleep problems	insomnia	13
Kidney problems	Frequent urination	5
	Urinary incontinence	8
Respiratory problems	Dry cough	6
	Sputum cough	5
	Dry cough	5

**Table 2:** Educational interventions in the intervention group

Session number	Session description
**The first session**	It was on the nature of cancer, treatments, chemotherapy, and the importance of self-care. It was a group meeting of five 10-member groups, held by two faculty members of the Nursing Department and the research using the educational film method.
**The second session**	It was on the nutrition, gastrointestinal complications, and care measures to manage and reduce these complications. Given the individual differences in terms of nutrition and the variety of complications in different individuals, the session was held individually by two faculty members of the Nursing Department and the researcher using the face-to-face teaching method. At least one member of the patient’s family was required to attend the meetings.
**The third session**	It was on the educational program dealt with skin and hair complications and the care measures to manage and reduce these complications. It was a group meeting on the causes of fatigue, held by two faculty members of the Nursing Department and the researcher using the question-and-answer method.
**The fourth session**	It was about pain, fatigue, shortness of breath, and decreased quality of sleep. The session was held individually by two faculty members of the Nursing Department and the researcher using the face-to-face teaching method at the presence of the patient’s family member.
**The five session**	It was on the ways to promote patient adjustment with mental, psychological, social, and family issues. It was held as a group meeting by a faculty member of the Psychiatry Department and the researcher through the use of the face-to-face teaching method. The control group received no intervention.

The self-care ability was measured by the experimental and control groups before and 2 months after the intervention.

**Fig. 1:**
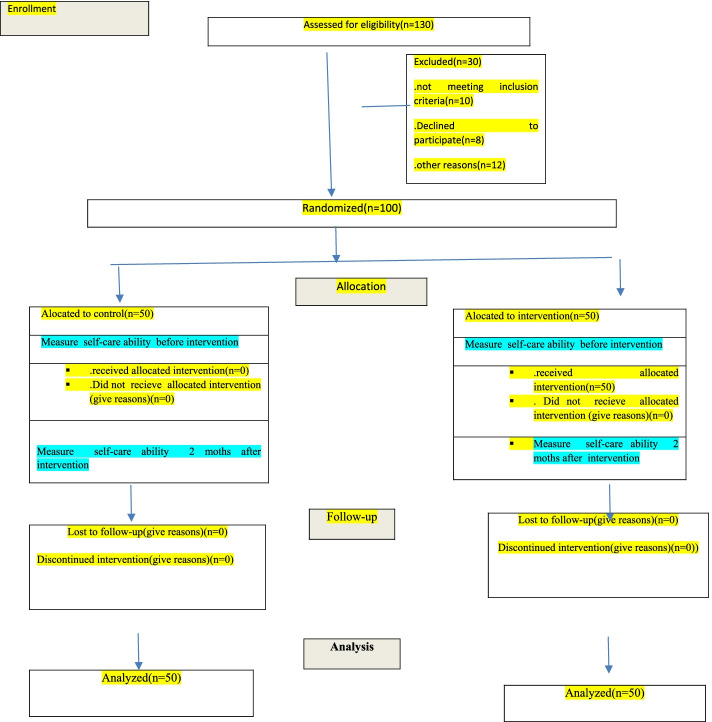
Flow Chart of Study

The educational methods used in this study included lectures, group discussions and questions and answers, film screenings. At the end of each training session, an educational booklet or booklet and a CD of educational videos related to the topic of the same session were provided to the intervention group. In addition to these cases, the researcher answered the questions of the intervention group both in person (attending the hospital chemotherapy unit) and by phone (the researcher’s telephone number was provided to the intervention group) and through the formation of an online social group, Supported the better implementation of the program. It should be noted that the researcher in the implementation of his educational program benefited from the cooperation of three faculty members of the nursing group (two members of the internal surgery group and one member of the psychiatric nursing group). No intervention was performed in the control group during the study. Two months after the implementation of the program, the self-care ability questionnaire was completed again for both intervention and control groups. We divided the intervention group into groups of 10 to 15 people and 5 training sessions for each group. After the intervention, the researchers had two face-to-face follow-up sessions. They then continuously sent self-care messages via WhatsApp group to the intervention group members, and if they had any questions, they would raise them in WhatsApp group and the experts would answer their questions. Two months after the implementation of the program, the self-care ability questionnaire was completed again for both intervention and control groups (Fig. 1).

### Instruments

In this study were used Self-care ability and the assess and identify the conceptual pattern of Orem questionnaires.

1. Self-care ability questionnaire

Self-care ability was the outcome measured in this study through the use of the Self-Care Inventory. The questionnaire had 40 items and was scored based on a 6-point Likert scale as follows: strongly agree (1), slightly agree (2), agree (3), disagree (4), slightly disagree (5) and strongly disagree (6). The scores ranged from 40 to 240, with the minimum score of 40 indicating a more desirable status of self-care ability and the maximum score of 240 indicating poor and inadequate self-care ability. The self-care ability scores were good (40–58), moderate (59–89), poor (90–160), and very poor (> 162). The questionnaires were localized in Farsi, and their validity and reliability had been evaluated and confirmed in several studies [25].

2. the assess and identify the conceptual pattern of Orem questionnaire

It was used in similar studies [26, 27]. The questionnaire consisted of four sections, the first of which included general health factors including age, sex, marital status, educational level, housing status, employment status, income and support of family members; The second part includes general self-care needs including air, water, food, defecation, activity / rest, socio-cultural interactions, health care system, family system, lifestyle pattern, environment and support system; The third part of self-evolutionary care needs includes the maintenance of the evolutionary environment, the prevention and maintenance of natural evolutionary threatening situations; The fourth section examines self-care needs in deviating from health, including adherence to the treatment regimen, attention to potential problems associated with the regimen, recording and modifying changes in health status, adapting to lifestyle, and adapting to changes in health status and diet regimen [28]. Based on this questionnaire, defects or deficiencies related to self-care were determined as nursing diagnoses.

### Data analysis

The data were analyzed using the SPSS20 software, so that the normality of the data was first assessed by skewness and kurtosis indexes. The values of skewness and kurtosis indexes between the ±2 interval were considered as the criterion for confirming the normality of univariate. Frequency, mean and standard deviation indices were used to describe the data, and paired t-test, independent t-test and chi-square test were also used for data analysis. The significance level was considered 0.05 in all tests.

## Results

The demographic and background information of the patients participating in this study is shown in Table 3. The mean and standard deviation of age in the control group was 35.06 ± 14.51, and 31.72 ± 15.01 in the experimental group was. The independent t-test showed no significant difference between the two groups in terms of age and body mass index. The Chi-square test also showed that there was no significant difference between the experimental and control groups in terms of gender, marital status, education, occupation, number of children, mood, insurance, support system, source of information, and language (*P* > 0.05).

**Table 3:** Frequency distribution of demographic information of the study participants

Variable		Control	Experimental	*p*-value
		n(%)	n(%)	
BMI		23.31 ± 5.03	23.20 ± 5.37	t (98) = .685 *p* = .410
Gender	Male	(70) 35	(70) 35	0.58
	Female	(30) 15	(30) 15	
Marital Status	Married	(74) 37	(68) 34	0.33
	Single	(26) 13	(32) 16	
Educated	Primary/Secondary	(68) 34	(48) 24	0.06
	Diploma	(28) 14	(36) 18	
	Academic	(4) 2	(16) 8	
Occupation	Housewife	(72) 36	(58) 29	0.44
	Employee	(10) 5	(14) 7	
	Unemployed	(4) 2	(10) 5	
	Student	(14) 7	(18) 9	
Number of Children	One	(38) 19	(52) 26	0.57
	Two or more	(62) 31	(48) 24	
Mood	Calm	(94) 47	(94) 47	0.71
	Anxious	(4) 2	(2) 1	
	Depressed	(2) 1	(4) 2	
Insurance	Yes	46 (92)	(94) 47	0.5
	No	(8) 4	(6) 3	
Support system	Spouse / Family	(66) 33	(76) 38	0.15
	Friends / acquaintances	(10) 5	(6) 3	
	Relief Foundation / Welfare Organization	(8) 4	(4) 2	
	Other	(14) 7	(14) 7	
Source of information acquisition	Client	(60) 30	(64) 32	0.44
	Family	(40) 20	(36) 18	
Language	Farsi	(46) 23	(50) 25	0.42
	Arabic	(54) 27	(50) 25	

The results of the independent t-test showed that before the intervention, there was no significant difference between the experimental and control groups in terms of the mean self-care (*P* = 0.38). But after the educational intervention, a significant difference was found between the two groups in terms of the mean self-care (*P* = 0.001) Table 4.

**Table 4:** Comparison of mean and standard deviation of self-care before and after intervention in two groups

Variable	Pre-intervention			Post-intervention		
	Experimental	Control	*P* value*	Experimental	Control	*P* value*
	Mean ± SD	Mean ± SD		Mean ± SD	Mean ± SD	
**Self-care**	6.78 ± 12.609	96.74 ± 26.10	0.001	98.54 ± 21.97	94.25 ± 40.21	0.38

Table 5 shows the mean and standard deviation of self-care in the experimental group before and after the intervention. After the educational intervention, the experimental group showed a significant improvement in the mean self-care score (*P* = 0.001), but no significant difference was observed in this variable in the control group (*P* = 0.06).

**Table 5:** Mean and standard deviation of self-care in experimental and control groups before and after intervention

Self-care	Post-intervention		Pre intervention		*p*-value
	Mean	SD	Mean	SD	
**Experimental group**	98.54	21.97	69.78	12.60	0.001
**Control group**	94.40	25.21	96.74	26.10	0.06

## Discussion

The present study aimed to evaluate the effect of education based on Orem’s self-care model on self-care ability of the patient’s undergoing chemotherapy, who referred to Shafa Hospital, Ahvaz city, iran in 2018.The results of this study showed that there was no significant difference in the mean self-care scores of the experimental and control groups before the educational intervention, but after the intervention, a significant difference was found between the two groups in terms of mean self-care. This confirms the effectiveness of Orem-based educational intervention on self-care in the patient’s undergoing chemotherapy. This is also in line with the results of various studies carried out in Iran [21, 29, 30] and other countries [31, 32] on cancer patients. Masodi et al. study was done on 70 multiple sclerosis patients. The experimental group was treated with 8 educational sessions about self-care based on Orem’s theory and was compared with the control group. The findings showed that after intervention a statistically significant difference was seen between the two groups and significant difference in experimental group between before and after self care performance, whereas, the same test showed no statistically significant difference in control groups [29]. This study was performed on patients with multiple sclerosis while the target population of the present study was cancer patients. In their study, Golchin et al. investigated the effect of the Orem model on the quality of life of cancer patients undergoing chemotherapy and obtained similar results [30]. In other words, there was no significant difference between the scores of the two groups before the intervention, but the difference was significant after the intervention [30]. This study was performed on patients with Acute Leukemia Receiving Chemotherapy while the target population of the present study was all All types of cancer patients were referred to the hospital. Karbaschi et al. studied the impact of Orem’s model-based self-care on the quality of life of military personnel suffering from cancer and undergoing chemotherapy, and obtained similar results. They stated that using Orem’s model-based self-care program with regard to the patients’ educational needs could affect their quality of life [24]. This study investigated the effect of education based on the Orem model on the quality of life of people with cancer, while the present study investigated the effect of education on self-care behavior of cancer patients.

In their study, Afrasiabifar et al. also investigated the effects of Orem’s model-based self-care on quality of life of the women with breast cancer who were undergoing chemotherapy, and obtained similar results with those of the present study [21]. This study investigated the effect of education based on the Orem model on the quality of life of people with breast cancer, while the present study investigated the effect of education on self-care behavior of all cancer patients. The results of this study showed that comparing the self-care status of the patients undergoing chemotherapy before the intervention (98.21 ± 54.97) and after that (94.25 ± 40.21) in the experimental group indicated a significant difference. The results of this part of the study are consistent with those of the studies by Aranda et al. [33], Gokal et al. [19], Zhang et al. [20], Mak et al. [34] and Rustøen T et al. [35]. The results of their studies showed that the educational program through providing self-care information increased the self-care ability of the cancer patients undergoing chemotherapy. Therefore, it is necessary for the nurses in cancer departments to educate cancer patients about self-care. Williams also found similar results in his study, and stated that self-care behaviors improved the lives of cancer patients [36]. Most of these studies have examined the effect of education based on the Orem model on self-care behavior of people with breast cancer, while the present study investigated the effect of education on self-care behavior in people with various cancers that Will have more comprehensiveness and generalizability. The results of the Orem’s model showed that the patients’ major need was the identification of their psychological problems, such as low self-confidence and high depression, which was consistent with the results of the study by Rainbird et al. that the greatest needs of cancer patients were related to psychological dimensions and medical information [37]. They especially complained about physical issues and fear of cancer expansion as well as family and social problems. In particular, patients with metastatic cancers had numerous physical, mental and social needs; therefore, the health care system is required to apply clear mechanisms in order to respond positively to the justified needs of such patients. Limitations of the present study include low sample size. Asking for free participation may be one of the reasons that led to the small number of samples in the present study. The use of self-reporting tools is another limitation of the present study Because the questionnaires of this study were filled out by the participants with the self-report method and considering that the measurement of the individual’s behavior with self-report Compared to more objective methods such as observation, it has lower validity and honesty. Another limitation of the present study is the study environment that cultural, social, or organizational factors of this environment may affect the results.

## Conclusion

In general, the results of the present study indicated that using Orem’s model-based self-care for cancer patients undergoing chemotherapy in the experimental group had a significant effect on increasing their self-care. Given that Orem’s model-based self-care is a non-pharmacological, non-invasive and low-cost method in controlling physical and psychological problems and can be easily educated to the patients and their families by nurses or family members, it is suggested that nurses in chemotherapy departments consider the Orem’s model-based comprehensive self-care program including comprehensive education and support for the clients as one of their main duties and help the clients by first examining their educational needs, which are in fact essential parts of the education process. This way, the patients might learn the ways to reach and maintain their maximum level of health, and their physical and psychological problems as well as the side effects might be reduced.
